# Post-Operative Delirium and Cognitive Dysfunction in Aged Patients Undergoing Cardiac Surgery: A Randomized Comparison between Two Blood Oxygenators

**DOI:** 10.3390/bioengineering10121429

**Published:** 2023-12-15

**Authors:** Lorenzo Mantovani, Elisa Mikus, Elena Tenti, Diego Sangiorgi, Samantha Zannoni, Andrea Cavallucci, Letizia Ferroni, Paolo Cimaglia, Valentina Tolio, Elena Tremoli, Carlo Savini

**Affiliations:** 1 Cardiovascular Department, Maria Cecilia Hospital, GVM Care & Research, 48031 Cotignola, Italy; lmantovani@gvmnet.it (L.M.); etenti@gvmnet.it (E.T.); dsangiorgi@gvmnet.it (D.S.); szannoni@gvmnet.it (S.Z.); acavallucci@gvmnet.it (A.C.); lferroni@gvmnet.it (L.F.); valentina.tolio@icloud.com (V.T.); etremoli@gvmnet.it (E.T.); csavini@gvmnet.it (C.S.); 2 Cardiology Unit, Azienda Ospedaliero Universitaria di Ferrara, 44124 Ferrara, Italy; paolocimaglia88@gmail.com; 3 Department of Experimental Diagnostic and Surgical Medicine (DIMEC), University of Bologna, 40126 Bologna, Italy

**Keywords:** cardiac surgery, cognitive function, oxygenators

## Abstract

In elderly patients undergoing cardiac surgery, extracorporeal circulation affects the incidence of post-operative delirium and cognitive impairment with an impact on quality of life and mortality. In this study, a new oxygenator system (RemoweLL 2) was tested against a conventional system to assess its efficacy in reducing the onset of postoperative delirium and cognitive dysfunction and the levels of serum inflammatory markers. A total of 154 patients (>65 y.o.) undergoing cardiopulmonary bypass (CPB) were enrolled and randomly assigned to oxygenator RemoweLL 2 (*n* = 81) or to gold standard device Inspire (*n* = 73) between September 2019 and March 2022. The aims of the study were to assess the incidence of delirium and the cognitive decline by neuropsychiatric tests and the MoCa test intra-hospital and at 6 months after CPB. Inflammation biomarkers in both groups were also evaluated. Before the CPB, the experimental groups were comparable for all variables. After CPB, the incidence of severe post-operative delirium showed a better trend (*p* = 0.093) in patients assigned to RemoweLL 2 (16.0%) versus Inspire (26.0%). Differences in enolase levels (*p* = 0.049), white blood cells (*p* = 0.006), and neutrophils (*p* = 0.003) in favor of RemoweLL 2 were also found. The use of novel and better construction technologies in CPB oxygenator devices results in measurable better neurocognitive and neurological outcomes in the elderly population undergoing CPB.

## 1. Introduction

In recent years, cardiac surgery approaches and techniques have undergone a very-important evolution and this has been made possible due to the growth of extracorporeal circulation (ECC) techniques and applications. ECC or cardiopulmonary bypass (CPB) was introduced for the first time in 1953 by Gibbon and today it is essential for almost all cardiac surgery [1]. The main function of the ECC is to replace the heart–lung function during the heart surgery procedure. Open-heart surgery requires temporarily stopping the heart and lungs and diverting the patient’s blood to an outside system that takes over the function of the heart and lungs. This is possible through the use of CPB, which diverts blood, through plastic tubing, to a heart–lung machine, which includes an oxygenator that works as an artificial lung. This allows for cardiac surgeons to operate in a field that is free of blood while the patient’s body continues to receive healthy blood.

It is now known that the use of ECC is associated with a systemic inflammatory state probably due to multifactorial causes such as contact between blood and artificial surfaces but also to the surgical intervention and/or to the release of endotoxins due to leukocyte activation [2]. The systemic inflammatory response is primarily sustained by activation of the classical and alternative pathways of the complement cascade, resulting in increased platelet aggregation and marked neutrophilic leukocyte adhesion [3]; moreover, recently, neuron-specific enolase and S100 calcium-binding protein β have been identified as possible serum biomarkers of post-operative delirium [4].

Despite improvements in the biocompatibility of CPB circuits, the activation of inflammatory systemic response may result in clinically relevant organ dysfunction [5]. Concerning the brain, prolonged hypoperfusion and microembolization during conventional CPB have been related to post-operative neurologic impairment with an incidence varying from 30% to 60% [6]. This clinical scenario covers a spectrum from a transient cognitive dysfunction to a permanent stroke. Post-operative delirium (POD) and post-operative cognitive dysfunction (POCD) are the perioperative disturbances of cognition that commonly affect older people undergoing cardiac surgery. Unlike other forms of delirium, POD usually results in early full recovery. POCD is more difficult to define and implies a temporal consciousness impairment associated with surgery [7], whereas POCD is characterized by impairment in attention, cognition, recognition, orientation, memory, and learning. It may result in prolonged hospitalization, increased morbidity and mortality, and a negative impact on quality of life after surgery [8,9,10,11,12].

Growing evidence suggests that the occurrence of neurological complications after cardiac surgery may be related to the characteristics of the CPB system. The device RemoweLL 2 (Eurosets, Medolla, Italy) has been developed and consists of the integration of a system to filter the leukocytes and a two-phase system to filter lipid micro-particles. This device has been designed to reduce the systemic inflammatory response and to reduce the embolization of lipid micro-particles. These effects should translate into a better outcome for the patient with less short and long term complications [9,13].

Thus, the aim of this study is to compare the performance of the newly developed RemoweLL 2 oxygenator with a standard oxygenator on the onset of POD. Secondary objectives were to estimate the effects on POCD and on serum indicators of inflammation and organ function.

## 2. Materials and Methods

### 2.1. Patients

The prospective randomized study was approved by the local Institutional Review Board and Ethics Committee (prot. 5080/2019 I.5/115). Patients were assigned to either the control arm or the treatment arm in a fixed allocation ratio of 1:1 without stratification, using a sequential block design with a block size of 4, allowing for constant balancing throughout the enrolment. The two groups were similar for age, sex, weight, body surface area, and blood components at baseline. The study included 154 subjects aged ≥65 year that were scheduled for cardiac surgery with use of CPB at the Cardiac Surgery Department of Maria Cecilia Hospital, Italy, between September 2019 and March 2022. The cardiac surgery with ECC included coronary artery bypass graft (CABG) and concurrent valve replacement, or double valve replacement. Exclusion criteria were chronic coagulopathies (spontaneous International Normalized Ratio INR > 2), dialysis treatment, previous ischemic/haemorrhagic cerebral events, tumors or immunological diseases, liver cirrhosis, decompensated diabetes, severe preoperative anaemia, and documented history of cognitive impairment estimated by a preoperative Mini-Mental State Examination (MMSE) value < 24 points.

Structured interviews were conducted preoperatively, post-operatively, and in follow-up visits at one and six months and one year after surgery. The assessment scales used were the MMSE for cognition, Katz staircase with Activities of Daily Living (ADL) and Instrumental Activities of Daily Living (IADL) for participants’ functional status, the Montreal Cognitive Assessment (MoCA) and the Trail Making Test for early detection of cognitive impairment [14], the Digit Span test for measure of verbal short term capability, and the Confusion Assessment Method (CAM) for brief mental status evaluations. For intubated patients, the non-verbal CAM ICU (Intensive Care Unit) version was performed [15]. The assessment of delirium was performed 3 times per day for the first 6 days after surgery.

Perfusion management was also carried out according to standardized protocols already in use in our center. ECC adjuvant materials were also homogeneous and standardized according to the best current standards. All patients, regardless of the randomization arm, were followed up clinically to 12 months after inclusion.

### 2.2. Measurements and Laboratory Data

Venous blood samples were obtained at three time points: before surgery (Pre-surg), 24 h after surgery (24 h), and three days after surgery (72 h). After blood centrifugation, serum was divided in aliquots and stored at −80 °C until analyzed. Inflammatory markers were detected by commercial kits: human Interleukin-6 (IL6) ELISA kit, human Interleukin-8 (IL8) ELISA kit, human TNF-Alpha ELISA kit, human FABP2 ELISA kit, human Cystatin C ELISA kit (purchased from Thermo Fisher Scientific, Waltham, MA, USA), human neuron-specific enolase ELISA kit, and human lipocalin-2 ELISA kit (purchased from Abcam, Cambridge, UK). Blood samples were harvested pre- and post-CBP filter for the quantification of white blood cells (WBC), platelets (PLT), neutrophils, lymphocytes, monocytes, eosinophils, basophils, and lipid micro emboli (LME). LME were stained as previously described [16]. Briefly, 1 mL of sample was gently mixed with 0.1 mL of 1% oil red O solution (Sigma-Aldrich, Burlington, MO, USA). The mixture was then allowed to mix gently for 15 min. The sample was centrifuged at 2500 rpm for 20 min at room temperature and supernatant was loaded into Thoma chamber [17]. LME, appearing as orange-red stained particles, were counted by a blind evaluator using a light microscope.

For acute respiratory distress syndrome (ARDS) evaluation, the lowest PaO_2_/FiO_2_ (the ratio of arterial oxygen partial pressure in mmHg to fractional inspired oxygen) during hospitalization was considered. Based on this value, ARDS severity was therefore categorized as no ARDS (PaO_2_/FiO_2_ > 300), mild (PaO_2_/FiO_2_ 201–300), moderate (PaO_2_/FiO_2_ 100–200), or severe (PaO_2_/FiO_2_ < 100) [18].

### 2.3. Blood Oxygenators

RemoweLL 2 is designed to reduce neurological injury and inflammatory response in patients undergoing cardiac surgery, thanks to its unique and patented cardiotomy features capable to reduce lipids and leukocytes coming from pericardial suction blood, it has a cardiotomy capacity of 800 mL, and it depletes lipids-leukocytes in two steps: a multilayer cascade filtration, for lipids and leukocytes, and a supernatant separator for lipids only). Data in the literature suggest that RemoweLL 2 technology is effective in the filtration of LME (Lipid Micro Emboli) in the clinical setting and the subsequent attenuation of NSE (neuron-specific enolase) release, a known marker of neurologic injury [19].

**Figure 1:**
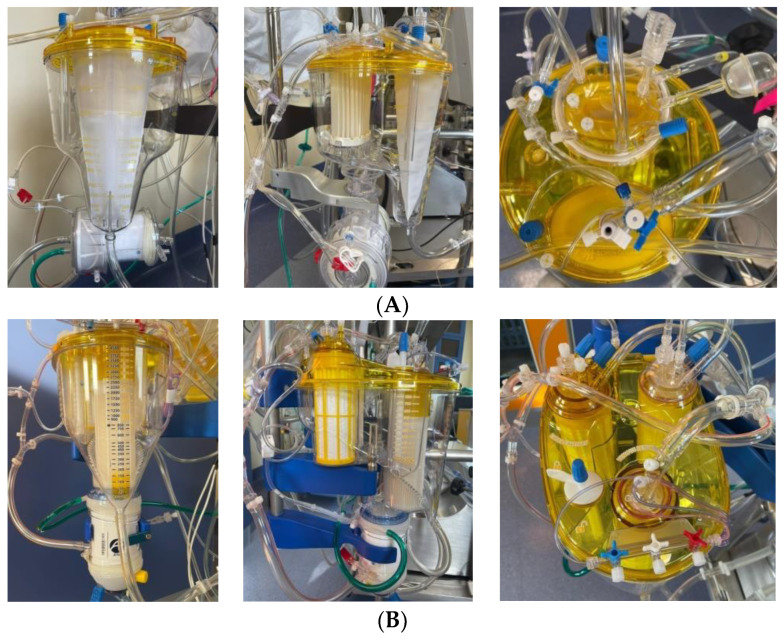
RemoweLL 2 and Inspire oxygenators. (**A**) Inspire: frontal view, lateral view with filter detail, chamber top view; (**B**) RemoweLL 2: frontal view; lateral view with filter detail; dual chamber top view.

Inspire has a 41 micron cardiotomy filtration which enables effective debris management; a venous filter dual screen also contributes to effective air management [20] (Figure 1).

### 2.4. Statistical Analysis

Continuous variables were reported as median and 1st–3rd quartile and compared with the Mann–Whitney test; categorical variables were reported as absolute number and frequencies and compared with Fisher’s exact test (1-sided for primary endpoint because of superiority hypothesis). For binary outcomes, logistic regression models were performed, and model discrimination was assessed using c-statistic. Count data were modelled using Poisson regression models. Repeated measures outcomes (cognitive and lab tests) were modelled using generalized linear mixed-effects models with appropriate link functions and families, depending on each single parameter distribution; robust standard errors were applied as appropriate. Anscombe residuals were analyzed for normality, influential observations were identified and excluded from models, and marginal effect plots were reported. All cognitive decline models comparing the two devices were adjusted for MMSE. Kaplan–Meier curves and log-rank test were reported for events at 1 year of follow-up. All analysis were performed with STATA 17.0 SE (StataCorp LLC, College Station, TX, USA); *p*-values < 0.05 were considered statistically significant.

## 3. Results

Overall, the study included 154 patients with a median age 76 years, 57% male. The most frequent comorbidities were mainly related to cardiovascular conditions: hypertension (86.8%), dyslipidemia (38.6%), peripheral artery disease (25.8%), type 2 diabetes (25.7%); depression and anxiety disorder were present in 6.6% and 2.6% of patients, respectively. Aortic valve surgery was performed on 73.2% of patients, mitral valve surgery on 51.3%, tricuspid surgery on 18.8%, and CABG on 70.8%, with a median CPB time equal to 106 min. No differences were observed between the two oxygenator systems (Table 1).

### 3.1. Mental Assessment and Cognitive Impairment

Percentages of patients with at least one positive CAM did not differ between Inspire and RemoweLL 2 (32.9 ± 10.8% vs. 34.6 ± 10.4%, respectively, *p* = 0.480), even if patients on Inspire had a higher percentage of positive CAMs (7.2 ± 1.6% vs. 5.9 ± 1.4%, *p* = 0.257) and a higher percentage of visits in which delirium therapy was prescribed (3.7 ± 1.1% vs. 2.9 ± 0.9%, *p* = 0.277). Delirium therapy at discharge was administered to 11.0 ± 7.2% vs. 6.2 ± 5.2%, respectively (*p* = 0.219). Considering a combined indicator of severe delirium, which takes into account both a higher (4+) number of positive CAMs or the administered delirium therapy (during hospital stay or at discharge), a positive trend in favor of RemoweLL 2 was observed (Table 2).

**Figure 2:**
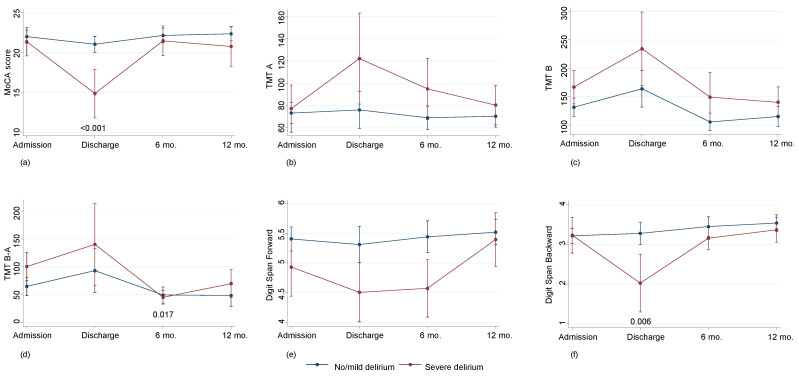
Cognitive decline according in patients with no/mild (*n* = 122) or severe (*n* = 32) delirium: Montreal Cognitive Assessment (MoCA) score (**a**); Trail Making Test (TMT) A (**b**); TMT B (**c**); TMT B-A (**d**) Digit Span Forward (**e**); Digit Span Backward (**f**). Values are predicted values and confidence intervals.

No difference in cognitive decline was observed in a direct comparison of oxygenators; nevertheless, comparing delirium severity, a cognitive impairment was observed both at discharge and at 6 months of follow-up but not at 12 months of follow-up (Figure 2).

In the overall group of patients, severe delirium showed a higher incidence during follow-up as compared to no/mild delirium: death log-rank *p* = 0.022, cerebrovascular event log-rank *p* = 0.012. Outcomes, however, did not differ between the two oxygenators: cardiovascular death log-rank *p* = 0.641, cardiovascular hospitalization log-rank *p* = 0.785, cerebrovascular event log-rank *p* = 0.993, death log-rank *p* = 0.822, any hospitalization log-rank *p* = 0.539, categorized ARDS *p* = 0.949.

### 3.2. Inflammatory Variables

**Figure 3:**
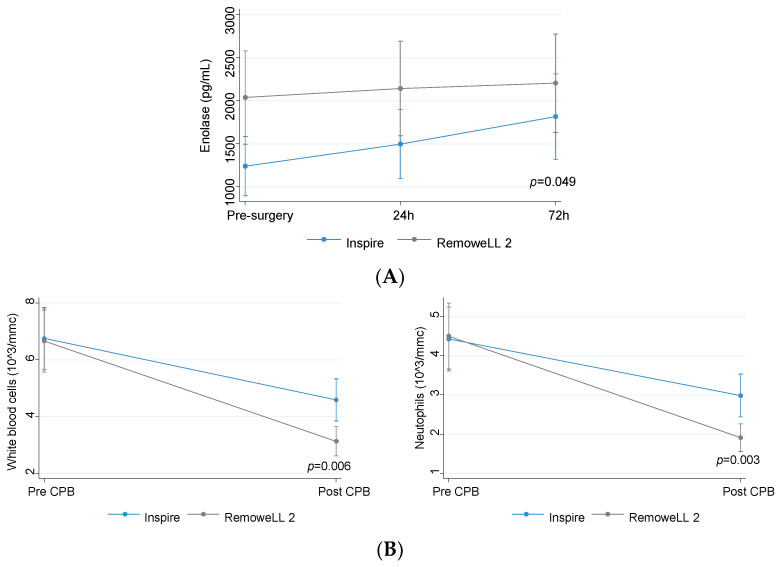
(**A**) Levels of enolase (predicted values and confidence intervals) in patients assigned to Inspire (*n* = 73) or RemoweLL 2 (*n* = 81) oxygenator; (**B**) levels of blood leukocytes in patients assigned to Inspire (*n* = 73) or RemoweLL 2 (*n* = 81) oxygenator. Total blood leukocytes (predicted values and confidence intervals) (left panel), neutrophils (predicted values and confidence intervals) (right panel).

The comparison of plasma levels of laboratory variables in the two group of patients showed a marked increase in enolase levels in patients of the Inspire group, which reached statistical significance 72 h after surgery (*p* = 0.049). Instead, no change in the levels of enolase occurred in RemoweLL 2 patients at all the time points assessed (Figure 3A).

White blood cells and neutrophils showed a greater, statistically significant (*p* < 0.01) decrease in RemoweLL 2 as compared to Inspire patients (Figure 3B).

**Figure 4:**
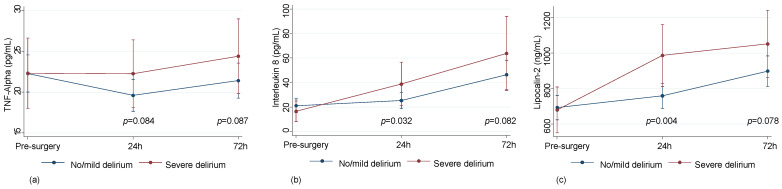
Levels of inflammatory markers in patients with no/mild (*n* = 122) or severe (*n* = 32) delirium. Plasma levels of TNFa (predicted values and confidence intervals) (**a**); Plasma levels of Interleukin 8 (predicted values and confidence intervals) (**b**); Plasma levels of Lipocalin-2 (predicted values and confidence intervals) (**c**).

Severe delirium was associated with rising trends in the serum levels of inflammatory markers, e.g., TNF-Alpha (*p* = 0.084 at 24 h and *p* = 0.087 at 72 h), Interleukin-8 (*p* = 0.032 at 24 h and *p* = 0.082 at 72 h), and Lipocalin-2 (*p* = 0.004 at 24 h and *p* = 0.078 at 72 h) (Figure 4) in all studied subjects.

No differences were observed between the two oxygenators in the all other inflammatory markers or cell variables (Interleukin-6, Interleukin-8, TNF-Alpha, FABP2, Cystatin C, lipocalin-2, LME, platelets, lymphocytes, monocytes, eosinophils, basophils).

## 4. Discussion

In this study carried out on patients undergoing aorto-coronary bypass surgery on a CPB, the RemoweLL 2 oxygenator was shown to be capable of reducing the extent of post-operative delirium with respect to a standard oxygenator. The use of RemoweLL 2 markedly reduced the reinfusion of lipid microemboli, which has been associated with a lower release of NSE into the bloodstream [21]. Instead, no difference was found between the two groups in the occurrence of delirium.

Of particular interest is the fact that the management pattern of delirium in the two treated groups (RemoweLL 2 vs. Inspire) differed appreciably. Patients of the RemoweLL 2 group experienced post-CPB delirium requiring less drug therapy, both during hospitalization and at discharge, compared to the patients of the Inspire group. We decided to use the Inspire in the comparison population as it represents the method routinely used in our center. According to the literature data [19,22], the results in terms of bubble removal are comparable to other oxygenators such as Fusion or Quadrox. To our knowledge, there are no studies of this type comparing different filters.

In this study, delirium was treated using a standard protocol (initially with haloperidol and in cases of persistence with intravenous dexmedetomidine infusion) and both groups of patients received the same treatments. The RemoweLL 2-treated group of patients, however, required less support from the clinical nursing staff compared to the Inspire-treated group of patients. In addition, serum levels of enolase did not increase in the RemoweLL 2 group at 24 and 72 h after surgery, despite them being higher at baseline levels; however, this difference was not statistically significant (*p* = 0.286). The crucial point, in our opinion, is not the markers at baseline level but their increase over time. Enolase proved to be stable in patients treated with RemoweLL2 and this is evidence that preoperative marker levels are of less importance. The fact that some biomarkers were increased and others were not was probably due to their greater sensitivity. The inflammation panel is complex, so often not all biomarkers show the same trend. Furthermore, the population studied is probably too undersized to be able to check the biomarker’s trend.

Several studies have shown the occurrence of frequent incident delirium after cardiac operations in adult patients and this has been considered a form of brain dysfunction. Additionally, cardiac surgery is highly invasive, and a previous study has shown that brain injury biomarkers, such as NSE, increase during CPB [23].

Thus, it can be hypothesized that brain injury is caused by cardiac surgery and that delirium may occur in the post-surgical period as a consequence of depressed brain function.

NSE was chosen as a surrogate marker of neurologic function because serum levels of NSE are significantly associated with the post-operative neurocognitive outcome [24], whereas other markers such as S100 calcium-binding protein β have were shown to be unspecific and do not correlate with neurologic or neuropsychological outcome. Indeed, Rasmussen and colleagues [5] found a significant correlation between the increase in NSE after CPB and the change in cognitive function at the time of discharge.

### Study Limitations

Due to the COVID 19 pandemic, it was not possible to recruit the expected number of patients in the study. In addition, the COVID-19 pandemic has increased the percentage of mood disorders in the general population. The data reported here need to be confirmed in larger studies with an appropriate number of patients.

## 5. Conclusions

In conclusion, the data reported here, although not statistically significant, indicate a trend in favor of RemoweLL 2 in terms of a reduction in the intensity and duration of post-operative delirium in the immediate aftermath, with a reduced need for the active involvement of intensive care personnel with the patient. These data are in favor of the clinical safety of the new RemoweLL 2 system and suggest its non-inferiority with respect to the oxygenators currently available.

## Figures and Tables

**Table 1:** Demographic and clinical characteristics.

	Inspire	RemoweLL 2	Overall	*p*
*n*	73	81	154	
Male, *n* (%)	49 (57.0)	48 (56.5)	97 (56.7)	1.000
Age (median, Q1–Q3)	75 (71–79)	76 (71–79)	76 (71–79)	0.623
BMI (median, Q1–Q3)	27.0 (24.7–29.8)	26.0 (24.2–28.7)	26.3 (24.4–29.1)	0.333
Recent hospitalization, *n* (%)	9 (12.5)	12 (14.8)	21 (13.7)	0.815
Hypertension, *n* (%)	59 (83.1)	73 (90.1)	132 (86.8)	0.234
Dyslipidemia, *n* (%)	25 (34.7)	34 (42.0)	59 (38.6)	0.407
Type 2 diabetes, *n* (%)	21 (29.2)	18 (22.5)	39 (25.7)	0.360
Prior MI, *n* (%)	4 (5.6)	3 (3.7)	7 (4.6)	0.706
Prior PCI, *n* (%)	5 (6.9)	11 (13.6)	16 (10.5)	0.199
Recent PCI, *n* (%)	1 (20.0)	1 (9.1)	2 (12.5)	1.000
Prior CABG, *n* (%)	1 (1.4)	0 (0.0)	1 (0.7)	0.471
Prior heart surgery, *n* (%)	8 (11.1)	5 (6.3)	13 (8.6)	0.387
Peripheral artery disease, *n* (%)	18 (25.4)	21 (26.3)	39 (25.8)	1.000
COPD, *n* (%)	7 (9.9)	9 (11.3)	16 (10.6)	1.000
Anxiety disorder, *n* (%)	0 (0.0)	4 (4.9)	4 (2.6)	0.123
Depression, *n* (%)	3 (4.2)	7 (8.8)	10 (6.6)	0.334
Parkinson’s disease, *n* (%)	0 (0.0)	1 (1.2)	1 (0.7)	1.000
Euroscore II (median, Q1–Q3)	3.1 (1.8–6.0)	3.5 (2.5–6.1)	3.4 (2.3–6.1)	0.325
White blood cells (median, Q1–Q3)	7.2 (6.2–8.6)	7.2 (6.4–8.2)	7.2 (6.2–8.3)	0.496
Haemoglobin (median, Q1–Q3)	13.2 (11.9–14.4)	13.2 (12.5–14.3)	13.2 (12.3–14.3)	0.629
Platelets (median, Q1–Q3)	194.0 (156.0–229.0)	192.0 (171.0–234.0)	193.5 (167.0–231.0)	0.639
Neutophils (median, Q1–Q3)	4.8 (4.0–5.9)	4.8 (3.9–5.7)	4.8 (3.9–5.7)	0.732
Limphocytes (median, Q1–Q3)	1.7 (1.2–2.1)	1.5 (1.2–1.8)	1.6 (1.2–2.0)	0.203
Monocytes (median, Q1–Q3)	0.6 (0.5–0.7)	0.6 (0.5–0.7)	0.6 (0.5–0.7)	1.000
Creatinine (median, Q1–Q3)	1.0 (0.8–1.2)	1.0 (0.8–1.2)	1.0 (0.8–1.2)	0.841
Cholesterol (median, Q1–Q3)	151.0 (125.0–187.0)	147.5 (128.5–169.5)	150.0 (128.0–175.0)	0.198
Triglycerides (median, Q1–Q3)	103.0 (76.0–133.0)	101.5 (86.5–119.0)	102.0 (81.0–126.0)	0.859
HDL (median, Q1–Q3)	42.0 (36.0–51.0)	45.0 (38.0–53.0)	43.5 (37.0–52.0)	0.396
LDL (median, Q1–Q3)	83.4 (68.4–108.2)	79.4 (64.6–98.1)	81.7 (65.6–102.4)	0.104
Albumin (median, Q1–Q3)	4.3 (4.1–4.5)	4.2 (3.9–4.4)	4.2 (4.0–4.4)	0.041
C Reactive Protein (median, Q1–Q3)	0.2 (0.1–0.4)	0.2 (0.1–0.6)	0.2 (0.1–0.5)	0.486
HS troponin (median, Q1–Q3)	16.5 (14.0–29.5)	17.0 (13.0–19.0)	17.0 (13.0–21.0)	0.596
CCS angina class, *n* (%)				0.905
1	4 (30.8)	6 (27.3)	10 (28.6)	
2	6 (46.2)	12 (54.5)	18 (51.4)	
3	3 (23.1)	4 (18.2)	7 (20.0)	
NYHA class, *n* (%)				0.203
1	3 (4.3)	4 (5.4)	7 (4.9)	
2	30 (43.5)	42 (56.8)	72 (50.3)	
3	36 (52.2)	27 (36.5)	63 (44.1)	
4	0 (0.0)	1 (1.4)	1 (0.7)	
Aortic valve surgery, *n* (%)	51 (69.9)	61 (76.3)	112 (73.2)	0.465
Mitral valve surgery, *n* (%)	41 (56.2)	38 (46.9)	79 (51.3)	0.263
Tricuspid surgery, *n* (%)	15 (20.5)	14 (17.3)	29 (18.8)	0.682
CABG, *n* (%)	48 (65.8)	61 (75.3)	109 (70.8)	0.217
CPB time, minutes (median, Q1–Q3)	109.0 (89.0–132.0)	102.5 (80.0–133.5)	106.0 (86.0–132.5)	0.252
Days to discharge (median, Q1–Q3)	11.5 (7.0–19.0)	13.5 (7.0–21.0)	12.5 (7.0–20.5)	0.500

Q1–Q3: 1st–3rd quartile; BMI: body mass index; MI: myocardial infarction; PCI: percutaneous coronary Intervention; CABG: coronary artery bypass grafting; COPD: chronic obstructive pulmonary disease; HDL: high-density lipoprotein; LDL: low-density lipoprotein; HS troponin: high-sensitivity cardiac troponin; CCS: Canadian Cardiovascular Society; NYHA: New York Heart Association; CPB: cardiopulmonary bypass.

**Table 2:** Assessment of CAM and delirium therapy.

	Inspire	RemoweLL 2	*p*
*n*	73	81	
Positive CAM out of total administered CAM	7.2% (70/971)	5.9% (64/1080)	0.257
Patients with ≥1 positive CAM	32.9% (24/73)	34.6% (28/81)	0.480
Patients with ≥4 positive CAM	12.3% (9/73)	6.2% (5/81)	0.148
Visits with delirium therapy out of total visits	3.7% (46/1240)	2.9% (40/1364)	0.277
Patients with delirium therapy at discharge	11.0% (8/73)	6.2% (5/81)	0.219
Patients with severe delirium, defined as: ≥4 positive CAM (>24 h) and/or delirium therapy in hospital/at discharge	26.0% (19/73)	16.0% (13/81)	0.093

## Data Availability

The data presented in this study are available on request from the corresponding author. The data are not publicly available due to data protection directive 95/46/EC.
